# Developing a Sealing Material: Effect of Epoxy Modification on Specific Physical and Mechanical Properties

**DOI:** 10.3390/ma6125490

**Published:** 2013-11-27

**Authors:** Christoph Schoberleitner, Vasiliki-Maria Archodoulaki, Thomas Koch, Sigrid Lüftl, Markus Werderitsch, Gerhard Kuschnig

**Affiliations:** 1 Institute of Materials Science and Technology, Vienna University of Technology, Favoritenstraße 9-11, Vienna 1040, Austria; E-Mails: vasiliki-maria.archodoulaki@tuwien.ac.at (V.-M.A.); thomas.koch@tuwien.ac.at (T.K.); s.lueftl@gmail.com (S.L.); 2 Vienna Water, Municipal Department 31, Grabnergasse 4-6, Vienna 1050, Austria; E-Mails: markus.werderitsch@wien.gv.at (M.W.); gerhard.kuschnig@wien.gv.at (G.K.)

**Keywords:** epoxy, modified epoxy, mechanical and physical properties, water influence

## Abstract

To develop a matched sealing material for socket rehabilitation of grey cast iron pipes, an epoxy resin is modified by the addition of different components to improve the flexibility. Three different modifications are made by adding ethylene-propylene diene monomer (EPDM) rubber powder, reactive liquid polymer (ATBN) and epoxidized modifier. In this paper the effect of the modification method as well as the influence of absorption of water on the mechanical and physical properties are analyzed in terms of: tensile strength, modulus of elasticity, adhesion performance, pressure resistance, glass transition temperature and water content. A comparison with neat epoxy shows for all materials that the modulus of elasticity and strength decrease. Unlike other tested modification methods, the modification with rubber powder did not enhance the flexibility. All materials absorb water and a plasticization effect arises with further changes of mechanical and physical properties. The application of the sealant on the grey cast iron leads to a reduction of the strain at break (in comparison to the common tensile test of the pure materials) and has to be evaluated. The main requirement of pressure resistance up to 1 MPa was tested on two chosen materials. Both materials fulfill this requirement.

## 1. Introduction

Developing Water Loss Prevention (DeWaLoP) is a project, led by Vienna Water, which includes a comprehensive concept for repairing leaky lead joint sockets. The sockets of the 150 year old grey cast iron pipes of Vienna’s water supplying system were caulked up to the 1920s with a hemp pack and a lead ring (M. Werderitsch, 25.08.2010, personal communication) and are to be replaced. 

The sealing was secured by a swollen hemp pack and a lead ring, stabilizing the hemp in the socket. A possible displacement of the lead ring and a decomposing of the hemp pack can cause leakage [1]. However, the static and metallurgical conditions of these grey cast iron pipes are not critical. Leakage leads to erosion of pipe bedding and, furthermore, imposes stress to the pipe, described by Burn and Rajani in [2,3]. As a result of this process, the likelihood of pipe breaks increases. Also, an economic loss in terms of damages to foundation of roads and buildings and to the water supply systems arises [2,4].

For this project, an in-pipe robot was designed, that crawls into water pipes (diameter 800 to 1000 mm), inspects, cleans and applies the sealing material [5]. The task covers a sealing material modification for pressure operating grey cast iron pipes. Such a special environment demands that numerous requirements and boundary conditions have to be met. The material has to be food-safe and fulfill the requirements of the national standard ÖNORM B5014-1:2012 [6] in order to eliminate a deterioration of the water quality. The operating pressure ranges from 0.4 to 0.6 MPa in supply systems. It depends on the difference in altitude. In the event of closing a gate valve in transport pipes, the pressure can increase up to 1 MPa (M. Werderitsch, 17.10.2012, personal communication). Because of inexact alignment during the assembly, rehabilitation problems could arise caused by a varying socket gap clearance. For a successful rehabilitation, a feasible socket gap is necessary. Thus, 5 to 30 mm gap clearance was set as boundary condition for the rehabilitation. Considering the above, the mechanical properties like stiffness and strength have to be sufficient. Also, appropriate elasticity has to be ensured to compensate for elongation due to minimal pipe movements.

Based on the previous investigations [7], epoxy materials have proved advantageous for this application. The thermoset materials form a rigid network having in large part proper material properties like pressure resistance, high stiffness and adhesion. Adversely, the high stiffness prevents the material capability to absorb relative movements between the pipe segments. Hence, the task is to develop a matched material formulation which combines toughness enhancement and good performance of the epoxy material. Therefore, in the recent work, epoxy resin is modified by the addition of different components to improve the flexibility. Hence, the addition of EPDM rubber powder (grain size 63–100 µm), reactive liquid polymer (RLP, amine-terminated butadiene-acrylonitrile copolymer) or epoxidized modifiers (flexibiliser with epoxy end-groups) is investigated. Also, two customized materials are tested. A comparison of the modifiers and their influence on mechanical and physical properties is discussed in this paper. Furthermore, the influence of water on the properties of the formulations is analyzed. The results are presented in Section 2, the experimental part is described in Section 3.

## 2. Results and Discussion

**Figure 1:**
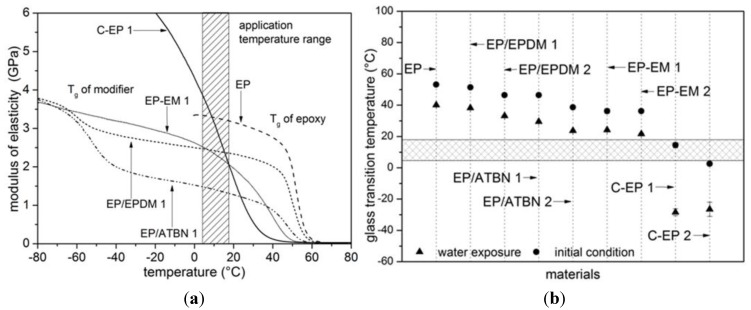
Results determined by dynamic mechanical analysis. (**a**) Storage modulus (Eʹ), examples of characteristic curves; (**b**) Change of glass transition temperature *T*_g_ due to water exposure for 28 days at 40 °C.

Figure 1a depicts exemplarily the modulus of elasticity measured by dynamic mechanical analysis. Modifications with reactive liquid polymer (EP/ATBN 1) or rubber powder (EP/EPDM 1) form a two-phase system. This reactive liquid polymer (ATBN) is miscible with epoxy resin. The functionalized chain ends like amine of the RLP can react with the epoxy phase and bond covalently to the epoxy resin. During curing, the rubber phase becomes less miscible with the resin, separates and form dispersed rubber particles in the matrix [8]. Due to the phase separation, this epoxy modification shows different glass transition temperatures (*T*_g_) of the modifier and the epoxy. Almost constant mechanical properties in the application temperature range and a decrease in the modulus of elasticity—in comparison to neat epoxy—can be observed. The material modification with epoxidized modifier (EP-EM 1) belongs to the group of internal plasticized epoxies. Epoxidized modifiers are added and dissolved as a second component in the resin. In the recent study, trifunctional modifiers are used which react with the curing agent and the modifying component will be incorporated in the epoxy matrix. The slope of the modulus of elasticity in the application temperature range of this modification is quite flat. The customized proprietary epoxy (C-EP 1) for sewer socket rehabilitation seems to be a copolymer of epoxy and flexibiliser (belongs to internal plasticization). These formulations show a strong slope of the modulus of elasticity in the application temperature range. Strong differences in the mechanical properties between minimum and maximum application temperature arise. 

Changes of the mechanical properties in the application temperature range are not desired. In order to avoid this, the *T*_g_ of the materials should not be in the same range as the application temperature. Modification of epoxy leads to a decrease of the *T*_g_ which is also reported in [9,10,11]. The dissolved rubber within the matrix of the formulations with the ATBN as well as the reduced crosslink density of the formulations with epoxidized modifiers cause the reduction of the *T*_g_.

In Figure 1b different modifier contents (e.g., EP/EPDM 1 and EP/EPDM 2) are compared. One can see that higher modifier content is leading to larger shift of *T*_g_ to lower temperatures. Using ATBN or rubber powder for modification, the materials show in addition to the *T*_g_ of the epoxy a second *T*_g_ of the rubber phase (Figure 1a). Reactive liquid polymer (formulation: 40 g modifier, 100 g resin) causes a greater reduction of the *T*_g_ than modification with rubber powder. An amount of 9 to 10% of epoxidized modifiers reduces the *T*_g_ significantly stronger than EPDM rubber powder. At the initial condition, the application temperature range and *T*_g_ overlap for the customized materials C-EP 1 and C-EP 2. In addition, the exposure of epoxy to water in any case results in a shift of the *T*_g_ to lower temperatures which is also shown by [12,13]. Consequently, the application temperature range is changed for each analyzed material. However, the shift of the *T*_g_ to lower temperatures enables a further consideration of the material C-EP 1.

**Figure 2:**
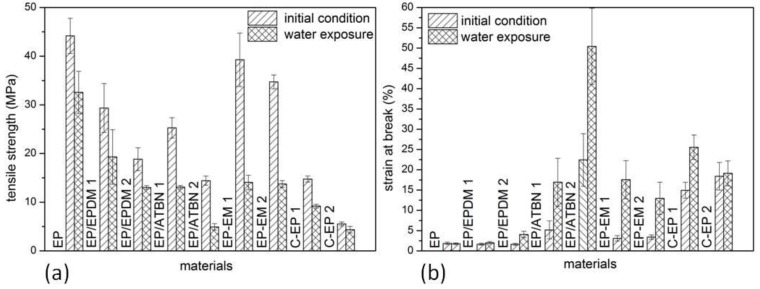
Results of the tensile test. (**a**) Tensile strength of the initial condition (fully cured) and of samples after water exposure for 28 days at 40 °C; (**b**) Strain at break of the initial condition (fully cured) and of samples after water exposure for 28 days at 40 °C.

Changes in the mechanical properties like modulus of elasticity and strength of flexibilized epoxy are also reported in [10,14,15]. The material behavior evaluated by means of tensile tests is shown in Figure 2a. The reduction by modification with the specified modifiers can reach more than 50% at the materials with the highest content of EPDM rubber powder and reactive liquid polymer. Otherwise, an amount of 9 to 10% of epoxidized modifiers influences the tensile strength less strongly as the same amount of rubber powder. The customized sealing materials show a low level of tensile strength. 

The main reason for the modification or flexibilization in the present work is the necessity of the materials ability to compensate relative movements between the pipe segments. These can be caused by temperature variations or subsidences in the pipe bedding. According to the results of the initial condition samples in Figure 2b, the most successful method seems to be the addition of reactive liquid polymer (EP/ATBN) or expoxidized modifiers. Chikhi explained the changes of the mechanical properties in [10] as follows: rubber affects the tensile properties and they depend on the compability of the rubber with the epoxy matrix, on the intrinsic strength of the rubber and on the rubber content. The decrease of the tensile strength is a consequence of ATBN addition, specifically might due to the effect of the softy structure of ATBN in the matrix. As described in [14] rubber particles are known to enhance the strain at break because of an interaction of the crack tip stress field with the rubber particles. The rubber particles cavitate and they initiate or assist yielding in the epoxy matrix. 

A precondition for the explanation above is a chemical reaction between the epoxy and the ATBN. Infrared spectroscopy is a valuable method to investigate the occurrence of chemical reactions between the epoxy resin and ATBN. However, it should be noted that ATBN hide the effect of the curing agent because both containing amine groups which react with the epoxy. Furthermore, Chikhi described in [10] that an increase of strain at break as well as a decrease of glass transition temperature of the epoxy can be an indication for chemical reactions between the ATBN and the epoxy. Both could be observed in the recent study. 

It is generally known that polymeric materials absorb water [16]. Epoxy as a polymeric material also absorbs water and this water absorption depends on crosslink density, exposure- temperature and time. Water disrupts the interchain Van der Vaals forces and results in an increase of chain mobility. So it acts like a plasticiser [17,18]. As it can be seen in Figure 2a,b, the tensile properties are strongly influenced by the water absorption of the materials. The tensile strength decreases and the strain at break increases. According to these results, the materials blended with rubber powder (EP/EPDM) are not suitable for this purpose.

**Figure 3:**
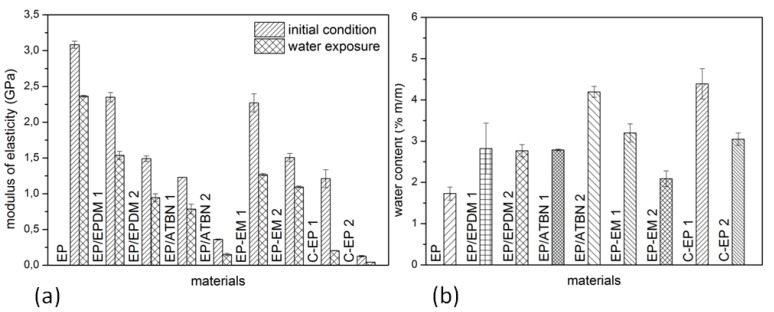
(**a**) Modulus of the initial condition (fully cured) and after water exposure for 28 days at 40 °C, dynamic mechanical analyses (DMA); (**b**) Water content of the materials after 28 days at 40 °C, investigated by thermogravimetric analysis (TGA).

In comparison to the neat epoxy, a reduction of the modulus of elasticity occurs at all formulations (Figure 3a). Due to the presence of the rubbery phase, the modulus of elasticity of the cured epoxy polymer decreases, probably due to lowering the crosslink density. Reactive liquid polymer, EP/ATBN (e.g., formulation: 40 g modifier, 100 g resin) influences the decrease of the modulus of elasticity more significantly than rubber powder, EP/EPDM. Comparing the formulation with two different epoxidized modifiers, the triglycidyl ether of propoxylated glycerol (EM 2) is more effective with its reduction of the modulus of elasticity up to 50% (in comparison to the neat epoxy) than the glycidyl ether of castor oil (EM 1). For all materials, a further reduction of the modulus of elasticity can be observed after water exposure for 28 days at 40 °C. The modulus of elasticity of material C-EP 2 is on a very low level and additionally influenced by water exposure. Hence, the material is not suitable for application and was rejected. The material C-EP 1 is highly influenced by water as well but further considered for application.

As it can be seen in Figure 3b no material is resistant to water absorption (determined by thermogravimetric analysis). The neat epoxy shows the lower water absorption from all formulations. The modification EP/ATBN 2 and the customized epoxy C-EP 1 show the highest water absorption of about 4.5% m/m. This corresponds well with their strong decrease of the mechanical properties like modulus of elasticity and strength, enhancement of strain at break as well as the shift of the *T*_g_. 

**Figure 4:**
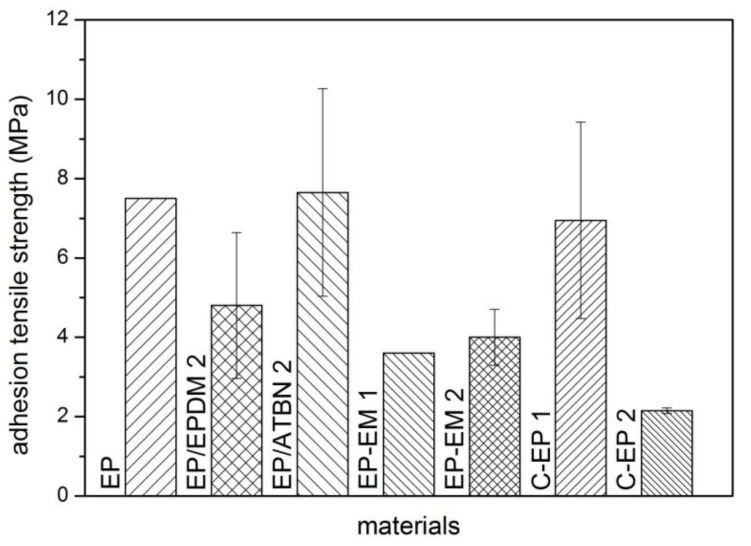
Adhesion tensile strength of the materials, initial condition.

The material properties gathered from tensile test differs significantly from the properties of the sealant applied on the grey cast iron. Therefore, adhesion tensile tests of the materials for the given purpose of the application are necessary and the results are shown in Figure 4. 

Adhesion tensile strength of neat epoxy is in the same range than those of epoxy modified with ATBN and the customized epoxy C-EP 1. The remaining materials show a reduction of their adhesion tensile strength.

**Figure 5:**
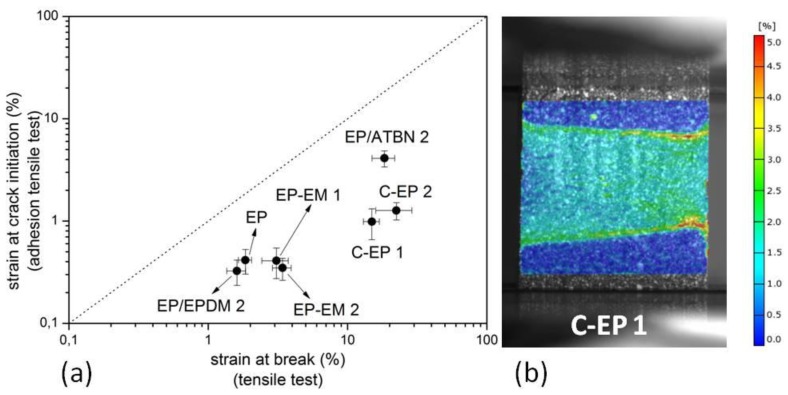
(**a**) Correlation of strain at break measured by common tensile test and strain at crack initiation from adhesion tensile test; (**b**) 2D-image correlation: Major strain at crack initiation of an adhesion tensile test sample, E-CP 1.

Figure 5a depicts the correlation between the strain at break (gathered from tensile test) and major strain at crack initiation of the grey cast iron-epoxy compound (described in 3.3). Processed as compound, no material could reach the strain at break measured by common tensile test. In this context, the differences in the cross-section of the samples (tensile test, adhesion tensile test) have to be noted. An increase of sample cross-section leads to a decrease of strain at break in a common tensile test. Nevertheless, the differences were not as high as in the adhesion tensile test. As shown in Figure 5b, it becomes clear that the strain distribution in the epoxy layer is inhomogeneous. Especially in the interphase near the adhesion surface, the strain is much higher than in the bulk, which results in crack initiation and failure at lower loads. The materials C-EP 1, C-EP 2 and EP/ATBN 2 achieved the best results.

**Figure 6:**
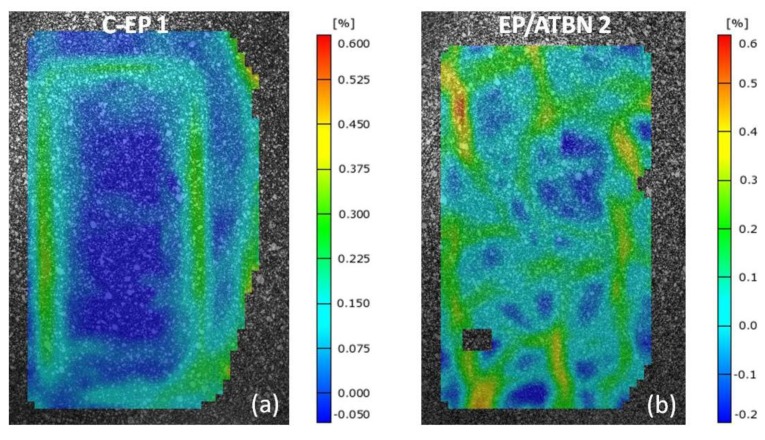
Major strain distribution, testing plate with 30 mm slot at a pressure of 1 MPa. (**a**) Material C-EP 1; (**b**) Material EP/ATBN 2.

Not only is the performance of the material in a compound with grey cast iron important, but the ability to compensate the pressure due to water in the pipe is also of great interest. Concerning the appropriate results of the materials EP/ATBN 2 and the customized epoxy C-EP 1 in the adhesion tensile test, the pressure resistance of these materials was investigated. At both, 10 and 30 mm slot all two materials fulfill the criterion of pressure resistance up to 1 MPa. The major strain is at both samples on a negligible level as can be seen in Figure 6a,b. The current study of the pressure resistance does not account the effect of water absorption by the materials. Considering the water influence in the pressure test, especially on the material C-EP 1, further investigations have to be done. 

## 3. Experimental Section 

### 3.1. Materials

The used base epoxy system is a proprietary but commercial available material. It is commonly used for pipe rehabilitation with an inliner, which means the in-pipe application of a needled felt liner impregnated with a thermoset material. The liquid resin and hardener used in this work for all modifications were:

- A diglycidylether Bisphenol-A (DGEBA) resin mixed with the diluent 1,6-hexandiglycidylether. The epoxy equivalent is 200 (g/eq)
- A customized proprietary amine curing agent

The acronym for the virgin, cured epoxy is EP.

The modifiers used are:

- EPDM rubber powder, grain size of 63–100 µm and a hardness of 40 Shore A
- Reactive liquid polymer; system of an amine-terminated butadiene-acrylonitril (ATBN) copolymer, with 16% acrylonitril and an amine equivalent weight of 900 g/eq
- epoxidized modifier 1, (EM 1): glycidyl ether of castor oil (three epoxy end-groups), epoxy equivalent 656 is g/eq
- epoxidized modifier 2, (EM 2): triglycidyl ether of propoxylated glycerol (three epoxy end-groups), epoxy equivalent is 644 g/eq

For reason of comparability two customized proprietary epoxies (primarily applied in the field for sewer rehabilitation) were also investigated (acronyms: C-EP 1, C-EP 2).

### 3.2. Sample Preparation

For preparing the formulations with reactive liquid rubber (EP/ATBN) the resin and ATBN were mixed together for 15 min in a bleaker and stirred by hand. The stoichiometric calculated amount of curing agent was added followed by further mixing for 5 min. The formulations with the epoxidized modifiers (EP/EM) were prepared as followed: the resin and the epoxidized modifier were stirred by hand in a bleaker for five minutes. The calculated amount of curing agent was added to the resultant mixture followed by further mixing for 5 min. Silica as a thixotropic agent for better processing was added to the formulations before adding the curing agent. For the epoxy/EPDM rubber powder (EP/EPDM) blend the resin and EPDM rubber powder were stirred by hand to a homogenous mixture. Finally the curing agent was added. The acronyms of the tested samples and their compositions are shown in Table 1. 

Producing the characteristic samples for the mentioned methods, silicon molds were used. If needed, the surface was grinded (polished) to remove irregularities. 

**Table 1:** Acronyms for the epoxies and their composition.

Acronym	Resin (g)	Modifier	Modifier content (g)	Silica
EP/EPDM 1	100	EPDM	10	60 mL
EP/EPDM 2	100	EPDM	40	–
EP/ATBN 1	100	ATBN	20	60 mL
EP/ATBN 2	100	ATBN	40	60 mL
EP-EM 1	90	EM1	10	60 mL
EP-EM 2	90	EM2	10	60 mL
C-EP 1	customized epoxy 1			
C-EP 2	customized epoxy 2			
EP	neat epoxy, used for modification			

### 3.3. Methods

Tensile strength (σ_M_) and strain at break were determined according to ISO 527-1 [19] with a common tensile testing machine, Zwick & Roell, model Z050 (Zwick GmbH & Co. KG, Ulm, Germany. Each testing series consisted of six specimens of shape 5A. The elongation was first measured with an extensometer (Zwick & Roell, model multiXtens) at a test speed of 1 mm/min for determining the modulus of elasticity. Followed by cross head detection and 50 mm/min for measuring the tensile strength and strain at break. 

The mass change of a specimen as a function of time and/or temperature was determined by thermogravimetric analysis (TGA) with a TGA 2050 (TA Instruments, New Castel, DE, USA). Cured material (initial condition) was investigated first, then the material after exposure to water for 28 days at 40 °C was tested. The mass loss up to 200 °C was considered for determining the water content of the sample. The TGA experiments were carried out using a heating rate of 20 K/min, air atmosphere as purge gas with a flow of 100 mL/min, a sample mass of app. 50 mg and an alumina pan. These tests were performed from ambient temperature to 600 °C according the standard ISO 11358 [20].

Dynamic mechanical analyses (DMA) were performed to assess the glass transition temperature (*T*_g_) and the storage modulus (Eʹ). All samples were measured with an oscillatory frequency of 1 Hz, a heating rate of 3 K/min and an amplitude of 70 µm in 3 point bending mode (50 mm support span). The glass transition temperature was determined at the maximum of the loss modulus Eʹʹ. According to the standard ISO 6721-1 [21] the measurements were arranged with a DMA 2980 (TA Instruments, New Castel, DE, USA). Sample dimensions are 10 mm × 3 mm × 60 mm (width × height × length).

**Figure 7:**
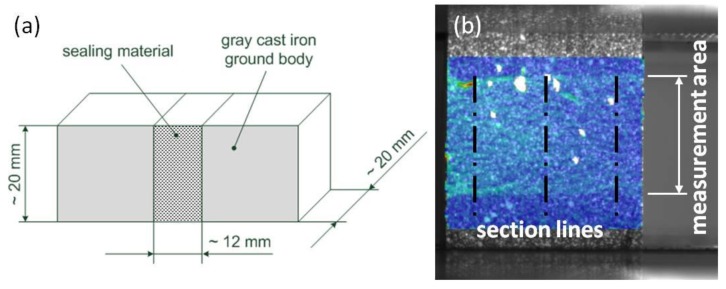
(**a**) Built up of the adhesion tensile test samples; (**b**) Scheme for determining the major strain at crack initiation from 2D-image correlation analysis.

Adhesion tensile tests were arranged to gather information about the materials adhesion properties at the grinded grey cast iron surface. The experiments were performed using a Zwick & Roell, model Z050 machine. The dimensions and the design of the samples are shown in Figure 7a. Test speed was 5 mm/min and the adhesion tensile strength was registered. For measuring the strain, a 2D-image correlation analysis was applied (GOM, Aramis). A dot pattern was applied on the samples and the recording frequency was 4 Hz. As strain at break, the major strain at crack initiation was analyzed as the medium of three sections through the measurement area (Figure 7b).

**Figure 8:**
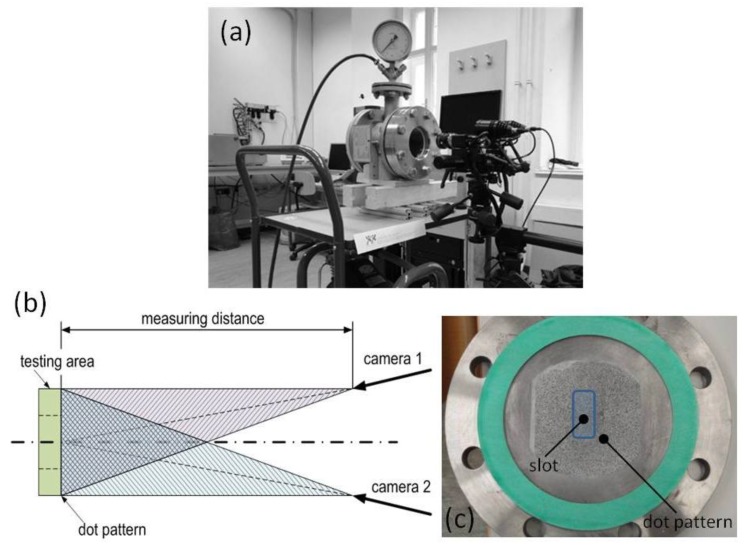
(**a**) In-house developed pressure chamber; (**b**) Scheme of the measurement setup for the 3D-image correlation with Aramis (GOM); (**c**) Testing plate with 30 mm slot and dot pattern.

The in-house developed pressure chamber test setup (Figure 8a) for the pre-testing of materials pressure resistance consists of three main components: a pressure chamber, an air compressor system, and a 3D-image correlation system. At the front side of the pressure chamber an inspection glass and at the backside the testing plate is mounted. The testing plate (Figure 8c) with its thickness of 22 mm has a 65 mm long slot. For analyzing the influence of the gap size on the materials pressure resistance, the widths of the slots in the testing plate are 10 mm or 30 mm. The maximum obtainable pressure is 1 MPa and pictures are taken second by second by two inclinated cameras (scheme of the experimental setup: Figure 8b) at room temperature. The 3D-image correlation analysis was arranged with Aramis (GOM) and the major strain is shown. 

## 4. Conclusions

The main reason for the modification or flexibilisation of an epoxy sealing material in the present work is the necessity of the materials’ ability to compensate relative movements between the pipe segments. Epoxy modified with reactive liquid polymer (ATBN), EPDM rubber powder and epoxidized modifiers as well as two customized epoxies were analyzed. The results show a comparison of the different methods to enhance the flexibility: Concerning the DMA results, nearly constant mechanical properties in the application temperature range (7–17 °C) could be observed at the formulations with reactive liquid polymer (ATBN) or EPDM. The customized proprietary epoxy and formulations with epoxidized modifier showed a slope in this temperature range. At all formulations a reduction of the mechanical properties like tensile strength and modulus of elasticity was observed. No enhancement of strain at break could be achieved by modifying with EPDM rubber powder. The glass transition temperature decreased and thus a change of the materials operating temperature range occurred. TGA measurements showed that all epoxy absorb water after immersion. Due to the water absorption, a plasticization effect was observed. Further changes in the materials operating temperature ranges have to be considered.

Mechanical properties evaluated by tensile tests could not be reached in the adhesion tensile test. Inhomogeneous strain distribution in these adhesive compounds and a maximum of stress near the interphase were leading to crack initiation at lower loads. The modified epoxy EP/ATBN 2 as well as the customized epoxy C-EP 1 fulfilled the requirement of pressure resistance up to 1 MPa. The measured major strain was negligible. Modification with ATBN is the most effective method of epoxy flexibility increase for this application.
